# Bis(2,4-dichloro­phen­oxy­acetato-κ^2^ *O* ^1^,*O* ^1′^)(5,5′-dimethyl-2,2′-bipyridine-κ^2^ *N*,*N*′)cobalt(II)

**DOI:** 10.1107/S1600536811010579

**Published:** 2011-03-26

**Authors:** Li-Li Ji, Jian-She Liu, Wen-Dong Song

**Affiliations:** a Environment Science and Engineering, Dong Hua University, Shanghai 200051, People’s Republic of China; b College of Science, Guangdong Ocean University, Zhanjiang 524088, People’s Republic of China

## Abstract

In the title compound, [Co(C_8_H_5_Cl_2_O_3_)(C_12_H_12_N_2_)], the Co^II^ atom, lying on a twofold rotation axis, is coordinated by four O atoms from two chelating 2,4-dichloro­phen­oxy­acetate ligands and two N atoms from a 5,5′-dimethyl-2,2′-bipyridine ligand, displaying a distorted octa­hedral geometry. A three-dimensional supra­molecular structure is formed through inter­molecular C—H⋯O hydrogen bonds and π–π stacking inter­actions between the pyridine and benzene rings [centroid–centroid distance = 3.779 (2) Å].

## Related literature

For related structures with 2,4-dichloro­phen­oxy­acetate ligands, see: Liu (2010 ▶); Song & Xi (2006 ▶).
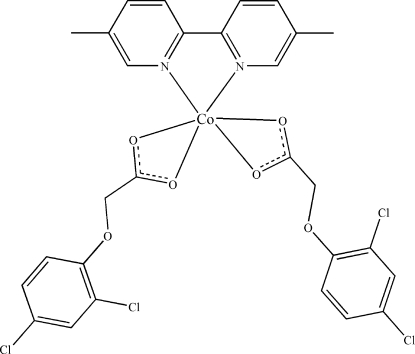


## Experimental

### 

#### Crystal data

[Co(C_8_H_5_Cl_2_O_3_)(C_12_H_12_N_2_)]
*M* *_r_* = 683.21
Monoclinic, 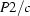

*a* = 13.397 (2) Å
*b* = 8.4152 (14) Å
*c* = 13.752 (2) Å
β = 112.570 (3)°
*V* = 1431.7 (4) Å^3^
*Z* = 2
Mo *K*α radiation
μ = 1.02 mm^−1^
*T* = 296 K
0.30 × 0.28 × 0.21 mm

#### Data collection

Bruker APEXII CCD diffractometer
Absorption correction: multi-scan (*SADABS*; Sheldrick, 1996 ▶) *T*_min_ = 0.756, *T*_max_ = 0.819
10343 measured reflections
2567 independent reflections
2229 reflections with *I* > 2σ(*I*)
*R*_int_ = 0.028

#### Refinement

*R*[*F* ^2^ > 2σ(*F* ^2^)] = 0.034
*wR*(*F* ^2^) = 0.106
*S* = 1.14
2567 reflections
187 parameters
H-atom parameters constrained
Δρ_max_ = 0.28 e Å^−3^
Δρ_min_ = −0.37 e Å^−3^

### 

Data collection: *APEX2* (Bruker, 2007 ▶); cell refinement: *SAINT* (Bruker, 2007 ▶); data reduction: *SAINT*; program(s) used to solve structure: *SHELXS97* (Sheldrick, 2008 ▶); program(s) used to refine structure: *SHELXL97* (Sheldrick, 2008 ▶); molecular graphics: *SHELXTL* (Sheldrick, 2008 ▶) and *DIAMOND* (Brandenburg, 1999 ▶); software used to prepare material for publication: *SHELXTL*.

## Supplementary Material

Crystal structure: contains datablocks I, global. DOI: 10.1107/S1600536811010579/hy2417sup1.cif

Structure factors: contains datablocks I. DOI: 10.1107/S1600536811010579/hy2417Isup2.hkl

Additional supplementary materials:  crystallographic information; 3D view; checkCIF report

## Figures and Tables

**Table 1:** Hydrogen-bond geometry (Å, °)

*D*—H⋯*A*	*D*—H	H⋯*A*	*D*⋯*A*	*D*—H⋯*A*
C7—H7*B*⋯O3^i^	0.97	2.49	3.391 (3)	154
C12—H12⋯O2^ii^	0.93	2.40	3.157 (4)	139

Symmetry codes: (i) 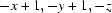; (ii) 
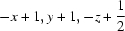
.

**Fig. 1.**
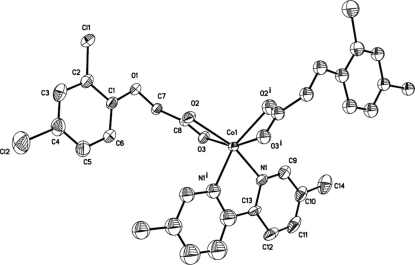
The molecular structure of the title compound, showing 50% probability displacement ellipsoids. [Symmetry code: (i) 1-x, y, 1/2-z.]

**Fig. 2.**
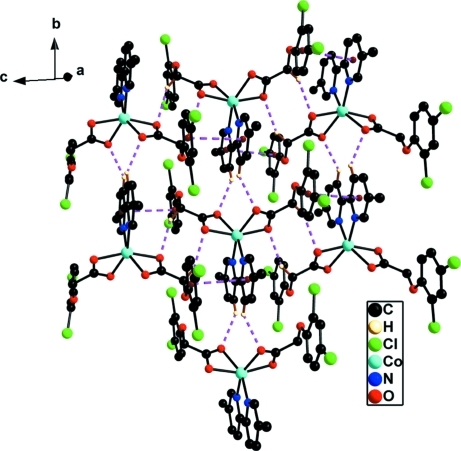
Packing diagram of the title compound. C—H···O hydrogen bonds and π–π interactions are shown as dashed lines. H atoms not involved in hydrogen bonds have been omitted for clarity.

## supplementary crystallographic information

### Comment

In the structural investigation of 2,4-dichlorophenoxyacetate complexes, it has been found that 2,4-dichlorophenoxyacetic acid functions as a multidentate ligand (Liu, 2010; Song & Xi, 2006), with versatile binding and coordination modes. In this paper, we report the crystal structure of the title compound, a new Co(II) complex obtained by the reaction of 2,4-dichlorophenoxyacetic acid, 5,5'-dimethyl-2,2'-bipyridine and cobalt nitrate hexahydrate in an alkaline aqueous solution.

As illustrated in Fig. 1, the Co^II^ atom, lying on a twofold rotation axis, exists in an distorted octahedral environment defined by four carboxylate O atoms from two different bidentate 2,4-dichlorophenoxyacetate ligands and two N atoms from one 5,5'-dimethyl-2,2'-bipyridine ligand. Intermolecular C—H···O hydrogen bonds (Table 1) and π–π stacking interactions are observed (Fig. 2). The centroid–centroid distance between a pyridine ring and a benzene ring at (1-*x*, 1-*y*, -*z*) is 3.779 (2) Å, thus indicating a weak π–π stacking interaction.

### Experimental

A mixture of 2,4-dichlorophenoxyacetic acid (0.110 g, 0.5 mmol), 5,5'-dimethyl-2,2'-bipyridine (0.092 g, 0.5 mmol), cobalt nitrate hexahydrate (0.145 g, 0.5 mmol), NaOH (0.08 g, 0.2 mmol) and H_2_O (10 ml) was placed in a 23 ml Teflon-lined reactor, which was heated to 423 K for 3 days, and then cooled to room temperature at a rate of 10 K h^-1^. The block purple crystals obtained were washed with water and dried in air (yield: 45% based on Co).

### Refinement

H atoms were positioned geometrically and treated as riding atoms, with C—H = 0.93 (atomatic), 0.96 (CH_3_) and 0.97 (CH_2_) Å and with *U*_iso_(H) = 1.2(1.5 for methyl)*U*_eq_(C). The hightest peak is located 0.86 Å from Cl1 and the deepest hole is located 0.73 Å from Cl1.

### Crystal data

**Table:**

[Co(C_8_H_5_Cl_2_O_3_)(C_12_H_12_N_2_)]	*F*(000) = 694
*M**_r_* = 683.21	*D*_x_ = 1.585 Mg m^−^^3^
Monoclinic, *P*2/*c*	Mo *K*α radiation, λ = 0.71073 Å
Hall symbol: -P 2yc	Cell parameters from 5837 reflections
*a* = 13.397 (2) Å	θ = 2.8–27.9°
*b* = 8.4152 (14) Å	µ = 1.02 mm^−^^1^
*c* = 13.752 (2) Å	*T* = 296 K
β = 112.570 (3)°	Block, purple
*V* = 1431.7 (4) Å^3^	0.30 × 0.28 × 0.21 mm
*Z* = 2	

### Data collection

**Table:**

Bruker APEXII CCD diffractometer	2567 independent reflections
Radiation source: fine-focus sealed tube	2229 reflections with *I* > 2σ(*I*)
graphite	*R*_int_ = 0.028
φ and ω scans	θ_max_ = 25.2°, θ_min_ = 1.7°
Absorption correction: multi-scan (*SADABS*; Sheldrick, 1996)	*h* = −16→16
*T*_min_ = 0.756, *T*_max_ = 0.819	*k* = −10→9
10343 measured reflections	*l* = −16→16

### Refinement

**Table:**

Refinement on *F*^2^	Primary atom site location: structure-invariant direct methods
Least-squares matrix: full	Secondary atom site location: difference Fourier map
*R*[*F*^2^ > 2σ(*F*^2^)] = 0.034	Hydrogen site location: inferred from neighbouring sites
*wR*(*F*^2^) = 0.106	H-atom parameters constrained
*S* = 1.14	*w* = 1/[σ^2^(*F*_o_^2^) + (0.0443*P*)^2^ + 1.0781*P*] where *P* = (*F*_o_^2^ + 2*F*_c_^2^)/3
2567 reflections	(Δ/σ)_max_ = 0.001
187 parameters	Δρ_max_ = 0.28 e Å^−^^3^
0 restraints	Δρ_min_ = −0.37 e Å^−^^3^

### Fractional atomic coordinates and isotropic or equivalent isotropic displacement parameters (Å^2^)

**Table:**

	*x*	*y*	*z*	*U*_iso_*/*U*_eq_	
C1	0.7163 (2)	0.1694 (3)	0.0470 (2)	0.0440 (7)	
C2	0.7908 (3)	0.0498 (4)	0.0494 (2)	0.0514 (7)	
C3	0.8966 (3)	0.0881 (5)	0.0701 (3)	0.0670 (10)	
H3	0.9462	0.0089	0.0732	0.080*	
C4	0.9289 (3)	0.2447 (5)	0.0861 (3)	0.0689 (10)	
C5	0.8566 (3)	0.3634 (5)	0.0807 (3)	0.0693 (10)	
H5	0.8790	0.4689	0.0900	0.083*	
C6	0.7503 (3)	0.3250 (4)	0.0612 (3)	0.0563 (8)	
H6	0.7013	0.4052	0.0577	0.068*	
C7	0.5307 (2)	0.2314 (3)	0.0072 (2)	0.0426 (6)	
H7A	0.4623	0.1799	−0.0321	0.051*	
H7B	0.5408	0.3142	−0.0373	0.051*	
C8	0.5240 (2)	0.3081 (3)	0.1036 (2)	0.0363 (6)	
C9	0.2940 (3)	0.6310 (4)	0.2236 (2)	0.0543 (8)	
H9	0.2643	0.5303	0.2203	0.065*	
C10	0.2281 (3)	0.7617 (5)	0.2140 (3)	0.0638 (9)	
C11	0.2754 (4)	0.9083 (5)	0.2190 (3)	0.0766 (12)	
H11	0.2344	0.9995	0.2134	0.092*	
C12	0.3815 (4)	0.9229 (4)	0.2320 (2)	0.0644 (10)	
H12	0.4123	1.0226	0.2343	0.077*	
C13	0.4424 (3)	0.7856 (3)	0.2417 (2)	0.0446 (7)	
C14	0.1122 (3)	0.7418 (6)	0.2007 (4)	0.0932 (14)	
H14A	0.0665	0.7517	0.1276	0.140*	
H14B	0.0937	0.8222	0.2404	0.140*	
H14C	0.1025	0.6387	0.2257	0.140*	
Cl1	0.74751 (8)	−0.14382 (10)	0.02104 (8)	0.0675 (3)	
Cl2	1.06334 (9)	0.28906 (19)	0.11140 (13)	0.1133 (5)	
Co1	0.5000	0.45028 (5)	0.2500	0.03575 (17)	
N1	0.3975 (2)	0.6418 (3)	0.23719 (18)	0.0429 (6)	
O1	0.61468 (16)	0.1179 (2)	0.02776 (17)	0.0482 (5)	
O2	0.59131 (17)	0.2812 (2)	0.19438 (15)	0.0471 (5)	
O3	0.44679 (16)	0.4039 (2)	0.08733 (15)	0.0460 (5)	

### Atomic displacement parameters (Å^2^)

**Table:**

	*U*^11^	*U*^22^	*U*^33^	*U*^12^	*U*^13^	*U*^23^
C1	0.0543 (17)	0.0405 (15)	0.0426 (15)	0.0081 (13)	0.0248 (13)	−0.0017 (12)
C2	0.0608 (19)	0.0482 (17)	0.0496 (17)	0.0134 (15)	0.0261 (15)	0.0016 (14)
C3	0.055 (2)	0.081 (3)	0.067 (2)	0.0212 (18)	0.0251 (17)	0.0001 (19)
C4	0.0500 (19)	0.084 (3)	0.074 (2)	−0.0019 (18)	0.0245 (17)	−0.013 (2)
C5	0.064 (2)	0.071 (2)	0.080 (3)	−0.0110 (19)	0.0352 (19)	−0.019 (2)
C6	0.061 (2)	0.0475 (18)	0.069 (2)	0.0053 (15)	0.0347 (17)	−0.0076 (16)
C7	0.0504 (16)	0.0378 (14)	0.0447 (15)	0.0063 (12)	0.0239 (13)	−0.0020 (12)
C8	0.0499 (15)	0.0237 (12)	0.0429 (15)	−0.0003 (11)	0.0262 (13)	0.0016 (11)
C9	0.0608 (19)	0.0546 (19)	0.0493 (17)	0.0114 (15)	0.0232 (15)	−0.0006 (14)
C10	0.079 (2)	0.067 (2)	0.0501 (19)	0.0319 (19)	0.0292 (17)	0.0086 (16)
C11	0.110 (3)	0.068 (3)	0.057 (2)	0.049 (2)	0.038 (2)	0.0093 (18)
C12	0.110 (3)	0.0374 (17)	0.0464 (18)	0.0194 (18)	0.0307 (19)	0.0019 (13)
C13	0.0789 (19)	0.0252 (13)	0.0302 (13)	0.0057 (12)	0.0217 (14)	−0.0008 (10)
C14	0.079 (3)	0.116 (4)	0.087 (3)	0.045 (3)	0.035 (2)	0.009 (3)
Cl1	0.0849 (6)	0.0432 (5)	0.0889 (7)	0.0162 (4)	0.0495 (5)	0.0002 (4)
Cl2	0.0555 (6)	0.1306 (11)	0.1526 (12)	−0.0124 (6)	0.0388 (7)	−0.0322 (9)
Co1	0.0512 (3)	0.0227 (3)	0.0400 (3)	0.000	0.0248 (2)	0.000
N1	0.0590 (15)	0.0341 (13)	0.0390 (13)	0.0066 (11)	0.0225 (11)	−0.0005 (10)
O1	0.0547 (12)	0.0372 (10)	0.0628 (13)	0.0063 (9)	0.0338 (10)	−0.0038 (9)
O2	0.0657 (13)	0.0352 (10)	0.0421 (11)	0.0102 (9)	0.0227 (10)	0.0026 (8)
O3	0.0540 (11)	0.0430 (11)	0.0456 (11)	0.0099 (9)	0.0242 (9)	−0.0030 (9)

### Geometric parameters (Å, °)

**Table:**

C1—O1	1.355 (4)	C9—H9	0.9300
C1—C6	1.375 (4)	C10—C11	1.377 (6)
C1—C2	1.408 (4)	C10—C14	1.500 (6)
C2—C3	1.374 (5)	C11—C12	1.368 (6)
C2—Cl1	1.723 (3)	C11—H11	0.9300
C3—C4	1.378 (6)	C12—C13	1.391 (4)
C3—H3	0.9300	C12—H12	0.9300
C4—C5	1.372 (5)	C13—N1	1.342 (4)
C4—Cl2	1.739 (4)	C13—C13^i^	1.470 (6)
C5—C6	1.383 (5)	C14—H14A	0.9600
C5—H5	0.9300	C14—H14B	0.9600
C6—H6	0.9300	C14—H14C	0.9600
C7—O1	1.419 (3)	Co1—N1^i^	2.080 (2)
C7—C8	1.508 (4)	Co1—N1	2.080 (2)
C7—H7A	0.9700	Co1—O3	2.1073 (19)
C7—H7B	0.9700	Co1—O3^i^	2.1074 (19)
C8—O2	1.249 (3)	Co1—O2	2.1963 (19)
C8—O3	1.262 (3)	Co1—O2^i^	2.1963 (19)
C9—N1	1.329 (4)	Co1—C8^i^	2.467 (3)
C9—C10	1.385 (4)		
			
O1—C1—C6	125.8 (3)	C13—C12—H12	120.7
O1—C1—C2	115.2 (3)	N1—C13—C12	120.5 (3)
C6—C1—C2	119.0 (3)	N1—C13—C13^i^	115.62 (16)
C3—C2—C1	120.2 (3)	C12—C13—C13^i^	123.9 (2)
C3—C2—Cl1	120.0 (3)	C10—C14—H14A	109.5
C1—C2—Cl1	119.8 (2)	C10—C14—H14B	109.5
C2—C3—C4	119.5 (3)	H14A—C14—H14B	109.5
C2—C3—H3	120.3	C10—C14—H14C	109.5
C4—C3—H3	120.3	H14A—C14—H14C	109.5
C5—C4—C3	121.1 (3)	H14B—C14—H14C	109.5
C5—C4—Cl2	120.6 (3)	N1^i^—Co1—N1	78.44 (14)
C3—C4—Cl2	118.3 (3)	N1^i^—Co1—O3	100.17 (8)
C4—C5—C6	119.5 (4)	N1—Co1—O3	96.32 (8)
C4—C5—H5	120.2	N1^i^—Co1—O3^i^	96.32 (8)
C6—C5—H5	120.2	N1—Co1—O3^i^	100.17 (8)
C1—C6—C5	120.7 (3)	O3—Co1—O3^i^	158.67 (11)
C1—C6—H6	119.7	N1^i^—Co1—O2	95.37 (9)
C5—C6—H6	119.7	N1—Co1—O2	155.51 (8)
O1—C7—C8	115.0 (2)	O3—Co1—O2	61.12 (7)
O1—C7—H7A	108.5	O3^i^—Co1—O2	104.07 (8)
C8—C7—H7A	108.5	N1^i^—Co1—O2^i^	155.51 (8)
O1—C7—H7B	108.5	N1—Co1—O2^i^	95.37 (9)
C8—C7—H7B	108.5	O3—Co1—O2^i^	104.07 (8)
H7A—C7—H7B	107.5	O3^i^—Co1—O2^i^	61.12 (7)
O2—C8—O3	121.4 (2)	O2—Co1—O2^i^	99.27 (11)
O2—C8—C7	122.5 (2)	N1^i^—Co1—C8^i^	126.52 (9)
O3—C8—C7	116.1 (2)	N1—Co1—C8^i^	98.99 (9)
N1—C9—C10	123.5 (3)	O3—Co1—C8^i^	132.83 (9)
N1—C9—H9	118.2	O3^i^—Co1—C8^i^	30.76 (8)
C10—C9—H9	118.2	O2—Co1—C8^i^	103.57 (8)
C11—C10—C9	116.2 (4)	O2^i^—Co1—C8^i^	30.36 (8)
C11—C10—C14	122.8 (3)	C9—N1—C13	119.5 (3)
C9—C10—C14	121.0 (4)	C9—N1—Co1	125.3 (2)
C12—C11—C10	121.5 (3)	C13—N1—Co1	115.1 (2)
C12—C11—H11	119.2	C1—O1—C7	118.9 (2)
C10—C11—H11	119.2	C8—O2—Co1	86.89 (15)
C11—C12—C13	118.7 (3)	C8—O3—Co1	90.57 (16)
C11—C12—H12	120.7		

Symmetry codes: (i) −*x*+1, *y*, −*z*+1/2.

### Hydrogen-bond geometry (Å, °)

**Table:**

*D*—H···*A*	*D*—H	H···*A*	*D*···*A*	*D*—H···*A*
C7—H7B···O3^ii^	0.97	2.49	3.391 (3)	154
C12—H12···O2^iii^	0.93	2.40	3.157 (4)	139

Symmetry codes: (ii) −*x*+1, −*y*+1, −*z*; (iii) −*x*+1, *y*+1, −*z*+1/2.
